# Treatment of chronic back pain by sensory discrimination training. A Phase I RCT of a novel device (FairMed) vs. TENS

**DOI:** 10.1186/1471-2474-9-97

**Published:** 2008-06-28

**Authors:** Karen L Barker, Christopher J Elliott, Catherine M Sackley, Jeremy CT Fairbank

**Affiliations:** 1 Physiotherapy Research Unit, Nuffield Orthopaedic Centre NHS Trust, Oxford, OX3 7LD, UK; 2 School of Health Sciences, University of Birmingham, Birmingham, B15 2TT, UK; 3 Department of Primary Care and General Practice, University of Birmingham Birmingham, B15 2TT, UK; 4 Nuffield Department of Orthopaedic Surgery, Nuffield Orthopaedic Centre, Oxford, OX3 7LD, UK

## Abstract

**Background:**

The causes of chronic low back pain (CLBP) remain obscure and effective treatment of symptoms remains elusive. A mechanism of relieving chronic pain based on the consequences of conflicting unpleasant sensory inputs to the central nervous system has been hypothesised. As a result a device was generated to deliver sensory discrimination training (FairMed), and this randomised controlled trial compared therapeutic effects with a comparable treatment modality, TENS.

**Methods:**

60 patients with CLBP were recruited from physiotherapy referrals to a single-blinded, randomised controlled, non-inferiority trial. They were randomised to receive either FairMed or TENS and asked to use the allocated device for 30 minutes, twice a day, for 3 weeks. The primary outcome variable measured at 0 and 3 weeks was pain intensity measured using a visual analogue scale averaged over 7 days. Secondary outcome measures were Oswestry Disability Index, 3 timed physical tests, 4 questionnaires assessing different aspects of emotional coping and a global measure of patient rating of change. Data were analysed for the difference in change of scores between groups using one-way ANOVA.

**Results:**

Baseline characteristics of the two groups were comparable. The primary outcome, change in pain intensity (VAS) at 3 weeks showed a mean difference between groups of -0.1, (non significant p = 0.82). The mean difference in change in ODI scores was 0.4; (non significant p = 0.85). Differences in change of physical functioning showed that no significant difference in change of scores for any of these test (p = 0.58 – 0.90). Changes in scores of aspects of emotional coping also demonstrated no significant difference in change scores between the groups (p = 0.14 – 0.94).

**Conclusion:**

FairMed was not inferior to TENS treatment.

The findings have implications for further research on current chronic pain theories and treatments. Further work to explore these mechanisms is important to expand our understanding of chronic pain and the role of neuro-modulation.

**Trial Registration:**

UKCRN Study ID 3321

## Background

Chronic low back pain (CLBP) is a very common symptom, and a major source of distress. The causes of back pain remain obscure and effective treatment of symptoms remains elusive. Most treatments are empirical, and few have withstood close scrutiny for effectiveness [1].

One approach is to understand the underlying causes, so that we can develop rational treatments. Current hypotheses of the basis of back pain can be broadly viewed as:

1. Biomedical or "Cartesian" models, where a pain sources or sources stimulate a pain pathway to the brain.

2. A bio psychosocial model.

This study arose because the senior investigator (JF) has become increasingly dissatisfied with Cartesian explanations for back pain, particularly following the MRC Spine Stabilisation Trial [2] where similar results were obtained in a Cartesian model (spinal fusion) and a non-cartesian model (intensive rehabilitation). In this light, the Harris hypothesis of conflicting sensory input is attractive [3], and lead to the development of a novel device to try to reduce this conflict.

Harris [3] proposed a hypothesis for a mechanism of chronic pain based on the consequences of conflicting sensory input to the central nervous system generating unpleasant sensations. He called this cortical pain. Flor et al [4] used a cortical pain model in a study that showed improving sensory discrimination for patients with intractable phantom limb pain led to cortical reorganisation and significant reductions in their pain.

Device treatment for pain has some obvious advantages over pharmaceutical treatments, particularly that nothing has to be ingested and the side effect profile is likely to be much less. It is also likely to be cheaper than pharmaceutical management and can be used in conjunction with those rehabilitation treatments for which there is evidence of efficacy. Device treatment is likely to have a potent placebo effect. There are many devices that purport to treat back pain. The current "market leader" is transcutaneous electrical nerve stimulation (TENS), estimated to be 1% of the analgesic market for back pain The treatment effect of TENS is attributed to influencing the "gate" proposed by Melzack and Wall in their Gate Control Theory of Pain [5]. TENS has been commercially successful. However, the clinical benefits of TENS remain controversial and there is lack of consensus regarding its efficacy. Some studies [6,7] suggest a lack of evidence to support its use in the treatment of chronic low back pain, while others found evidence of benefit, or have concluded that there is a lack of evidence of effect, rather than evidence of a lack of effect [8,9]. The recent Cochrane Review (2005) [6] concluded that there was limited or inconsistent evidence for TENS. However, no other device has any better evidence of efficacy and for this reason TENS was chosen as a comparator treatment for a new device (FairMed) that has been developed to deliver sensory discrimination training in patients with chronic back pain.

The aim of this study was to compare a novel sensory discriminatory training device (FairMed) with TENS in a Phase I clinical trial.

## Methods

### Trial design

The trial design was a single-blinded, longitudinal randomised controlled, non-inferiority trial.

Ethical approval was given by the Milton Keynes Local Research Ethics Committee (05/Q1603/34) and all patients were approached and gave informed consent to participate in the study.

### Sample size

A power calculation for a non-inferiority trial was made [10]. The non-inferiority limit (-d) was set at 20 on a 100 mm Visual Analogue Scale back pain scale as the primary outcome measure. A minimal clinical difference change of 20 mm was set [11]. From a pilot sample of 50 current patients the standard deviation (σ) of the VAS back was 2.07. δ = d/σ, δ = 0.966. This required 25 patients in each arm of the trial, allowing for 10% attrition/non-compliance rate, 55 subjects were required. This gave a 90% power and a Type 1 error rate of 2.5%.

### Participants

Patients referred to a Physiotherapy Department with a diagnosis of chronic low back pain were recruited to the study.

**Inclusion criteria **were patients aged over 18 years with a diagnosis of chronic low back pain lasting a minimum of 3 months.

**Exclusion criteria **were patients with leg pain, those who were current TENS users or patients where either of the treatment modalities might harm them. This was based on the warnings and precautions for the use of TENS namely, patients with pacemakers, damaged or broken skin, malignancy, poorly enervated areas and spinal infection.

### Randomisation

Randomisation was performed using a random numbers table. Randomisation was restricted to permuted blocks to ensure equal numbers being allocated to each group. Four blocks of patient assignment were used; two blocks of 0–9 and two of 0–19. Each random permuted block was transferred to a sequence of consecutively numbered, sealed, opaque envelopes and these were stored in a locked drawer until required. As each participant formally entered the trial, the researcher opened the next envelope in the sequence in the presence of the patient.

### Blinding

We attempted to ensure integrity of blind assessment by maintaining separation between the therapists providing the treatment and those assessing We followed practical tips to try and reduce 'unblinding' accepted within the field [12], such as participants being reminded before each assessment not to reveal details of their allocation group.

### Interventions

Patients were randomly allocated to use either TENS or the FairMed device. For the TENS group a dual channel portable TENS TPN 200 PLUS unit was used. Stimulation was based upon best available evidence from the literature. For conventional TENS, parameters between 80–100 Hz and 100–200 μs are considered to be effective in the treatment of chronic pain [6,9,13]. In studies specifically on chronic low back pain populations [7,9], parameters of 80 Hz/140 μs and 100 Hz/125 μs resulted in pain reduction. It is suggested that patients use TENS as much and as often as required [13]. Our protocol used stimulation given in continuous trains at high frequency (80 Hz, using square-wave 100 μs pulses). Two surface electrodes (5 cm × 5 cm^2 ^TPN 40 each) were placed in or adjacent to the painful area at a distance of 5 cm–20 cm apart. The intensity of TENS was adjusted to produce a tingling sensation that was approximately 2–3 times the sensory threshold.

**Figure 1:**
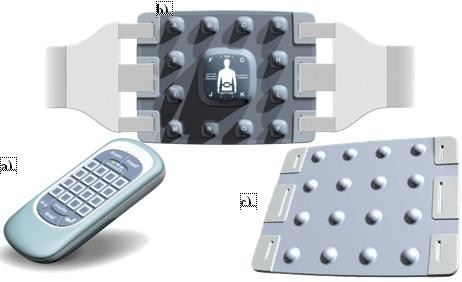
The FairMed; a) hand held controller and back array [b) posterior view and c) anterior view].

The FairMed group received the device (Figure 1) which includes 2 components: a hand held controller as subject interface and an array of 16 vibrators closely applied to the lumbar spine. One or more stimulator is activated randomly and the subject responds by indicating which stimulator(s) are active. The device display indicates if the response is correct or not via a visual and auditory display. The cycle is then repeated until the test session is complete. This requires a high level of attention. Subjects were asked to use the device for 30 minute sessions. The FairMed is specifically not a device to deliver either painful stimuli or massage. The stimulus is as localised as possible; it is brief and probably insufficient to impair position sense. The device was designed to teach localised discrimination of stimuli to the lumbar spine. The device was conceived by JF, developed in conjunction with KB, and prototype models were produced by a product design and development consultancy, which completed pre-trial laboratory testing.

### Outcome measures

The outcome measures used in this trial were collected in accordance with recommendations from the Initiative on Methods, Measurement, and Pain Assessment in Clinical Trials [IMMPACT]. These 6 core outcome domains are i) pain, ii) physical functioning, iii) emotional functioning, iv) participant ratings of improvement and satisfaction with treatment, v) symptoms and adverse events, and vi) participant disposition [14].

Pain was recorded using a 100 mm VAS recording patient's present pain intensity level, their average and worst pain intensity levels recorded over a week and averaged [15].

Physical functioning was recorded using the Oswestry Disability Index v 2.1 [16]. Functional physical tests – 5 minute walking distance, 1 minute stair climb and 1 minute standing up and sitting down from a chair were used to assess those aspects of physical performance most relevant to everyday activities [17].

Emotional functioning was assessed using a battery of questionnaires, the Health Anxiety and Depression Scale used to assess emotional functioning [18]; the Tampa Scale for Kinesiophobia (TSK) to assess pain-related fear of movement [19]; the Pain Coping Scale (PCS) to assess the three components of catastrophising: rumination, magnification and helplessness [20] and the Pain Self Efficacy Questionnaire to assess people's self-efficacy beliefs [21].

A global rating of patient reported improvement and satisfaction, the Patient Global Impression of Change scale [22], was assessed, having been widely used in chronic pain clinical trials e.g. Farrar, Young et al.[23], the PGIC has been demonstrated to have validity and it provides a responsive and readily interpretable measure. Participants were asked how they felt after their treatment, firstly, with regards to their ability to cope with their pain and, secondly, to their ability to perform everyday activities.

Participants completed outcome questionnaires at baseline, 3 weeks, 6 weeks and 12 weeks. They underwent functional testing at baseline and 3 weeks with a masked assessor. Over the 3 weeks that participants used their devices, they were asked to keep a daily pain diary.

Patients were explicitly asked about any adverse events they experienced using the devices, and to record these in their pain diary and by telephone report. An adverse event is considered any unwanted effect detected in participants of a clinical trial regardless of whether the effect can be attributed to the intervention under evaluation [24].

Finally, information on participant disposition was collected to document the recruitment of participants and their progression through the trial, numbers receiving intended treatment, those completing the study protocol and analysed for the primary outcome [14,24].

### Data analysis

Data were analysed using intention-to-treat analysis and the statistical package SPSS 12.0.1 for Windows.

Non-parametric statistics were used to compare outcome measures between groups (One way ANOVA). Statistical significance was set at the p < 0.05 level.

## Results

60 patients were randomised and recruited to the trial, 32 into the FairMed group and 28 into the TENS Group, 27 in each group completed the trial. Reasons for dropping out varied: one participant was admitted to hospital for spinal decompression surgery during his trial period, one participant withdrew citing personal circumstances, and four participants decided that they were unable to comply with the protocol after they had begun on the trial. None of the dropouts completed any re-assessments and so could not be used in further analysis.

**Figure 2:**
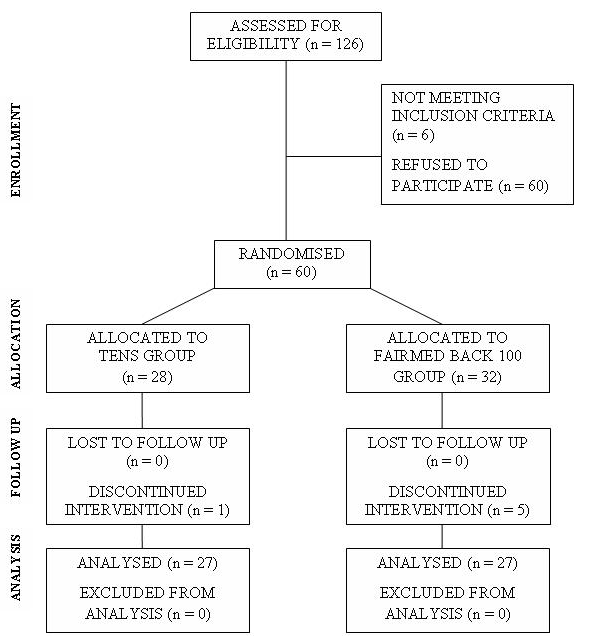
CONSORT diagram showing flow of participants through each stage of the trial.

Other than the 6 participants who withdrew before completion of the trial, there was no further loss to follow up. A CONSORT flow diagram is shown in figure 2.

### Participant characteristics

The mean age of participants was 53.4 years (SD 11.5). Both TENS and FairMed groups comprised equal percentages of men (50%) and women (50%). Analysis of all baseline characteristics demonstrated no significant difference between groups (Table 1).

**Table 1:** Participant Baseline Characteristics

**Treatment**		**Age (Years)**	**Average Pain Intensity (VAS 0–10)**	**Oswestry Disability Index (0–100%)**	**PSE (0–60)**	**TSK (17–60)**	**PCS (0–52)**	**Sit to Stand (N°/min)**	**Stair Climb (N°/min)**	**Walk Distance (metres/5 min)**
**FairMed**	**N**	32 (16 male; 16 female)	32	32	32	32	32	32	32	32
	**Mean**	52.7	6.3	40.8	31.0	40.4	19.6	13.7	6.5	235.6
	**Std. Deviation**	10.7	1.9	15.9	11.2	7.6	12.0	7.6	2.7	103.2
	**Range**	28 – 73	2 – 9	12 – 80	8 – 57	23 – 51	2 – 47	2 – 38	0 – 11.5	40 – 400
	**Median**	55	6	40	30	42	20.5	13	6	260
	**IQR**	47 – 59	5 – 8	30.5 – 47.5	21.8 – 37	34 – 46.8	10 – 26.5	8.5 – 16	5.3 – 8	160 – 300

**TENS**	**N**	28 (14 male; 14 female)	28	28	28	28	28	28	28	28
	**Mean**	54.1	6.6	42.8	27.6	41.3	23.0	12.7	6.7	219.6
	**Std. Deviation**	12.5	1.4	14.8	10.1	7.4	11.3	6.7	3.0	111.7
	**Range**	27 – 74	4 – 10	18 – 74	8 – 44	29 – 55	5 – 44	4 – 29	1 – 12.5	0 – 400
	**Median**	55	7	40.1	27	42	22	11	6.5	250
	**IQR**	42.5 – 65.8	5 – 7	30.8 – 53.5	21.3 – 36	34 – 46.8	14.3 – 28.8	9 – 17	4.5 – 9	100 – 320

#### Primary measure – pain intensity

Table 2 summarises the changes in outcome measures between baseline and 3 weeks. The primary outcome of the study concerned the participants' change in baseline pain intensity at 3 weeks. Worst, average and present pain intensity were all recorded and summaries of the raw data studied. The pattern of change in pain intensity scores was similar for each sub-category and as such, only the change in average VAS scores were analysed further. The mean difference in change of participants' average pain intensity VAS scores between those participants in the TENS group and those in FairMed group was -0.1., and was not statistically significant (p = 0.82).

**Table 2:** Change in outcome measures between baseline and 3 weeks – group differences.

**OUTCOME MEASURE**	**MEAN**	**SD**	**LOWER C.I. (95%)**	**UPPER C.I. (95%)**	SIGNIFICANCE*
**VAS (Average)**					
**FairMed**	-0.8	1.8	-1.5	-0.1	0.83
**TENS**	-0.7	1.4	-1.3	-0.1	
**PSE**					
**FairMed**	1.9	7.8	-1.0	4.8	0.21
**TENS**	4.4	7.5	1.5	7.4	
**TSK**					
**FairMed**	-1.8	5.8	-4.0	0.3	0.94
**TENS**	-2.0	7.7	-5.0	1.1	
**PCS**					
**FairMed**	-1.6	5.4	-3.6	0.4	0.84
**TENS**	2.0	7.7	-5.0	1.1	
**ODI**					
**FairMed**	-0.6	8.7	-3.8	2.7	0.85
**TENS**	-0.9	5.1	-3.0	1.1	
**HAD-A**					
**FairMed**	-1.4	2.7	-2.4	-0.4	0.14
**TENS**	-0.3	3.0	-1.4	0.9	
**HAD-D**					
**FairMed**	-0.2	2.0	-1.0	0.5	0.49
**TENS**	-0.6	1.9	-1.3	0.1	
**Sit to Stand**					
**FairMed**	1.0	2.1	0.1	1.8	0.90
**TENS**	1.0	2.2	0.1	1.9	
**Stairs**					
**FairMed**	0.4	1.9	-0.4	1.1	0.81
**TENS**	0.5	1.0	0.1	0.9	
**Walk Distance**					
**FairMed**	3.1	41.4	-12.6	18.9	0.58
**TENS**	9.1	39.2	-6.4	24.6	

SD (Standard Deviation).

C.I. (Confidence Interval).

VAS (Visual Analogue Scale).

PSE (Pain Self-Efficacy).

TSK (Tampa Scale for Kinesiophobia).

PCS (Pain Catastrophising Scale).

ODI (Oswestry Disability Index).

HADS-A (Hospital Anxiety & Depression Scale – Anxiety).

HADS-D (Hospital Anxiety & Depression Scale – Depression).

(* p-value from One-Way ANOVA on mean change scores across treatments).

#### Secondary measures

##### Disability (Oswestry Disability Index)

The mean difference in change in ODI scores of those participants in the FairMed and those in TENS groups was 0.4; this difference in change was not significant (p = 0.85).

##### Emotional functioning

No significant difference in change of scores of TSK, PSE, PCS and HAD were detected for any of these outcome measures.

##### Physical functioning

There were no significant differences in change of scores for physical functioning using the 1 minute sit to stand, 1 minute stair and 5 minute walk test scores at baseline and at 3 weeks.

##### Improvement and satisfaction ratings

In the FairMed group, 27% of participants stated that they were more able to cope with pain at 3 weeks; in the TENS group, this number was to 45%. However, 11% of TENS group participants also stated that they were less able to cope with pain at 3 weeks. 73% of FairMed group participants stated no change in their ability to cope with pain, compared to 44% in the TENS group.

## Discussion

The FairMed is a novel device and as such there are no existing data on effectiveness. It is difficult to establish what is a fair comparator to use in a trial with a new device. Whilst the Cochrane review of TENS concluded that there was limited evidence for TENS [6], there are no other devices that provide any better evidence of efficacy, nor any which have stood the test of time as well as TENS. For this reason TENS was chosen as a comparator treatment for a new device designed to help those patients with chronic back pain who continue to have serious difficulties, despite not being a treatment endorsed by current guidelines [1].

The findings of this trial demonstrate no significant difference in pain intensity reduction between the FairMed and TENS. This is in keeping with other trials investigating sensory changes, such as the findings of Guieu et al. [25] and Lundeberg [26] who also found no significant difference in pain reduction between vibratory stimulation and TENS in patients suffering with chronic musculoskeletal pain. Both trials used a control of a similar high frequency TENS and vibration protocol to the ones in the trial. There is a plethora of different outcome measures that can be used in the assessment of low back pain. In this study the physical outcome measures chosen were a walk test, timed stair climb and sit to stand. These physical tests have been advocated by other trial designs [17] and reported for reliability in a similar clinical population to the one in this study [27], hence the rationale for their use. However, it is possible that selection of physical performance measures that stressed the spine more specifically, such as loaded forward reach may have been more responsive to change in this patient population.

The theory for the analgesic effect of TENS is suggested to be as a result of counter stimulation of the nervous system modifying the perception of pain [5]. There is evidence to suggest that this same theory could be applied to the FairMed. Studies have demonstrated that superficial and deep mechanoreceptors exist with a high sensitivity to vibratory stimulation [26,28,29]. With a surface area of almost 500 cm^2^, the FairMed stimulates a large region of the lumbar spine and underlying tissues, indicating that pain alleviation could be attributed to the activation of these receptors.

Functional magnetic resonance imaging (fMRI) of subjects has revealed reduced activity in the anterior cingulated, insula and thalamus; some of the key pain processing areas of the brain. There is evidence to suggest that the same is true for cognitively demanding distraction. Bantick et al [30] reported decreased perception of painful stimuli in subjects receiving noxious thermal stimulus whilst engaged in a cognitively demanding task. Using fMRI, decreased activation in the same key components of the pain matrix have been reported [31]. Although these results are based on acute experimentally induced pain, modulation of chronic pain could conceivably be achieved by the FairMed through interference from vibratory stimulation and cognitive regulation of attention. Furthermore, Flor et al [7] demonstrated a reduction in phantom limb pain following a treatment programme using sensory discrimination training, demonstrated to be as a result of cortical reorganisation in the primary somatosensory cortex.

The study was severely limited by the functionality and reliability of the prototype FairMed device. Of the 32 devices used, 13 had developed faults by the end of the trial. Of the 32 participants randomised to the FairMed group, 20 reported some kind of fault with its functionality. Feedback from users of the FairMed device will be used to develop a more reliable device. It would have been preferable for this information to have been gathered before the main trial, but this was not possible due to time restrictions.

We recognise that this is a very difficult group to help and any treatment is likely to be of limited benefit. Commercially and practically it is difficult to demonstrate benefit in such chronic pain populations and very large studies are needed to show superiority. At the early stages in the development of a new and initially expensive device, a non-inferiority study is thus the only financially and practicably viable option. The main difficulties in the design and conduct of a non-inferiority trial are the demonstration of assay sensitivity, bias from lack of masking and difficulty in establishing the non-inferiority margin. As a consequence, the results of a non-inferiority trial are not as credible as those from a successful superiority trial [32,33]. However, a superiority trial requires a large positive mean difference and a small standard error of the mean difference to demonstrate a significant difference between interventions [33] and thus requires a much larger sample size [32,34]. Alternatively, in order to establish assay sensitivity using a non-inferiority trial design, it could be useful to include a placebo group in addition to the active control group [32]. However, given the relatively small difference hypothesised to exist between the FairMed and TENS, the sample sizes required to successfully demonstrate superiority or to include a third intervention group were considered prohibitive. Nevertheless, with greater resources, a more robust trial design could have been adopted.

## Conclusion

The findings presented in this study are not able to demonstrate a reduction in chronic low back pain using the FairMed. None-the-less, cortical reorganisation through sensory discrimination may be a mechanism through which the FairMed could modulate CLBP. Further work to explore these mechanisms is still needed to expand our understanding of chronic pain and the role of neuro-modulation in the transition from acute to chronic LBP.

## Competing interests

The authors declare that they have no competing interests.

Jeremy Fairbank has applied for a patent for the FairMed device and should the Fairmed device ever be marketed, he may benefit financially.

## Authors' contributions

JF and KB conceived, planned and secured funding for the study. CE collected all data and did the data analysis. CS supervised the data analysis and contributed to the manuscript preparation. KB co-ordinated the study and drafted the manuscript. All authors contributed to and approved the final manuscript.

## Pre-publication history

The pre-publication history for this paper can be accessed here:


